# Bromine as a Specific in Pseudo-Membraneous Affections

**Published:** 1856-10

**Authors:** 


*Cholera in Moscow.*

Cholera is raging fearfully in Moscow, which is said to be the true cause of the postponement of the coronation of the Czar.

